# Direct Interaction Between CD163 N-Terminal Domain and MYH9 C-Terminal Domain Contributes to Porcine Reproductive and Respiratory Syndrome Virus Internalization by Permissive Cells

**DOI:** 10.3389/fmicb.2019.01815

**Published:** 2019-08-06

**Authors:** Gaopeng Hou, Biyun Xue, Liangliang Li, Yuchen Nan, Lu Zhang, Kuokuo Li, Qin Zhao, Julian A. Hiscox, James P. Stewart, Chunyan Wu, Jingfei Wang, En-Min Zhou

**Affiliations:** ^1^Department of Preventive Veterinary Medicine, College of Veterinary Medicine, Northwest A&F University, Yangling, China; ^2^Department of Infection Biology, Institute of Infection and Global Health, University of Liverpool, Liverpool, United Kingdom; ^3^State Key Laboratory of Veterinary Biotechnology, Harbin Veterinary Research Institute, Chinese Academy of Agricultural Sciences, Harbin, China

**Keywords:** PRRSV, CD163, MYH9, protein–protein interaction, virus internalization

## Abstract

Porcine reproductive and respiratory syndrome virus (PRRSV) has a highly restricted tropism for cells of the monocyte-macrophage lineage, including porcine alveolar macrophages (PAMs). PRRSV entry into permissive cells involves several mediators in addition to two required host cell receptors, CD163 and MYH9. It is unknown whether CD163 directly interacts and/or cooperates with MYH9 to facilitate PRRSV infection. In this study, CD163 and MYH9 were co-immunoprecipitated from PAMs regardless of PRRSV infection status. Further truncation analysis indicated that the CD163 N-terminal region, containing scavenger receptor cysteine-rich domains 1 to 4 (SRCR1-4), directly interacts with the MYH9 C-terminal domain region without involvement of other adaptor proteins. Meanwhile, non-permissive HEK293T cells that stably expressed truncated swine CD163 SRCR1-4 domain did not support virus attachment. However, virus attachment to cells stably expressing SRCR5-CT domain was demonstrated to occur without appreciable virus internalization. The involvement of the SRCR1-4 domain in virus internalization was further demonstrated by the fact that incubation of recombinant SRCR1-4 protein with PAMs abolished subsequent virus internalization by permissive cells. These results demonstrated that CD163 SRCR1-4 interacts with the MYH9 C–terminal domain to facilitate PRRSV virion internalization in permissive cells, thus expanding our understanding of PRRSV cell-invasion mechanisms.

## Introduction

Porcine reproductive and respiratory syndrome (PRRS), an infectious disease that first emerged in the United States in 1987, currently causes huge economic losses to the swine industry worldwide (Nan et al., 2017). The clinical symptoms of PRRS include reproductive failure in pregnant sows and respiratory disorders in young piglets (Snijder et al., 2013). PRRS virus (PRRSV), the causative agent of PRRS, is a single-stranded positive-sense RNA virus (Das et al., 2010). The latest classification system classifies all PRRSV isolates into two species within the genus *Porartevirus*: PRRSV-1 and PRRSV-2 (Adams et al., 2016; Kuhn et al., 2016). PRRSV has a highly restricted cell tropism whereby it infects cells of the monocyte-macrophage lineage (Van Breedam et al., 2010) that including porcine alveolar macrophages (PAMs), the primary target of PRRSV infection *in vivo* (Rossow et al., 1995; Qi et al., 2017). Meanwhile, African green monkey kidney cell line MA-104 and its sub-clone MARC-145 are also susceptible to PRRSV infection and have been frequently used in PRRSV studies *in vitro* (Kim et al., 1993; Song et al., 2018).

The entry of PRRSV into permissive cells is mediated by numerous receptors or cellular factors, such as heparin sulfate (HS) (Delputte et al., 2002), vimentin (Kim et al., 2005), CD151 (Wu et al., 2014), CD163 (Guo et al., 2014), sialoadhesin (CD169) (Delputte et al., 2007), DC-SIGN (CD209) (Pineyro et al., 2016), and non-muscle myosin heavy chain 9 (MYH9) (Gao et al., 2016). Many studies have demonstrated that CD163 is an indispensable receptor for PRRSV infection, since the introduction of CD163 into non-permissive cell lines can confer susceptibility to infection (Calvert et al., 2007; Delrue et al., 2010; Wang et al., 2013; Li et al., 2017) and absolute resistance to PRRSV infection is observed in CD163 knock-out pigs (Whitworth et al., 2016; Burkard et al., 2017, 2018; Yang et al., 2018). Nevertheless, the detailed mechanism of how CD163 interacts or cooperates with other cellular components to achieve PRRSV entry into permissive cells is inadequately understood.

CD163 is a glycosylated transmembrane protein belonging to the scavenger receptor cysteine-rich (SRCR) family, consisting of a signal peptide, nine SRCR domains, two proline-serine-threonine (PST)-rich regions, a transmembrane domain and a cytoplasmic tail (Van Gorp et al., 2010). MYH9 belongs to the non-muscle myosin II heterohexamer, which is composed of four light chains and two MYH9 heavy chains, and the two heavy chains held together through coiled-coil rod domains (Li et al., 2018). MYH9 is involved in the cell migration, shape maintenance, and signal transduction (Liu et al., 2019). To date, available data from co-immunoprecipitation (co-IP) assays suggests that PRRSV glycoproteins (GP) GP2a and GP4 interact with CD163, with the last 223 carboxy-terminal amino acid (aa) residues of CD163 apparently uninvolved in this interaction (Das et al., 2010). By contrast, our previous studies identified non-muscle myosin heavy chain 9 (MYH9) as a cellular interaction partner for PRRSV-GP5 that is indispensable for PRRSV infection (Gao et al., 2016; Li et al., 2018). Notably, in MYH9-deficient non-permissive cell lines such as COS7, introduction of porcine CD163 is not sufficient to confer susceptibility to PRRSV without co-expression of MYH9 (Gao et al., 2016). In addition, it is still unclear whether CD163 and MYH9 cooperate with one another to facilitate PRRSV invasion. Meanwhile, another line of investigation involving the study of cell inflammatory responses demonstrated that a soluble form of CD163 co-localized with MYH9 within activated T lymphocytes (Timmermann et al., 2004), supporting a potential interaction between these two proteins. However, it remains unclear if a direct interaction between CD163 and MYH9 occurs during PRRSV infection and whether such an interaction is required to confer cell permissibility to PRRSV infection of permissive cells.

Here, we generated truncated constructs of CD163 and co-expressed both truncated CD163 proteins and the MYH9 C-terminal domain (PRA) in HEK293T cells to locate interacting domains within CD163 and MYH9. Co-IP assays suggested that CD163 SRCR1-4 domains, which were originally thought to be non-essential for PRRSV infection, interact with the MYH9 C-terminal domain to facilitate effective internalization of PRRSV virions by permissive cells. Moreover, the treatment of PAMs with the recombinantly expressed CD163 SRCR1-4 could significantly inhibit both PRRSV-1 and PRRSV-2 infections via competitive binding for MYH9 with the endogenous CD163. Ultimately, this study reveals a novel role played by CD163 and MYH9 in PRRSV infection and provides new insight into mechanisms involved in PRRSV pathogenesis.

## Materials and Methods

### Cells and Viruses

Porcine alveolar macrophages were prepared from 4- to 6-week-old PRRSV-negative pigs as previously described (Patel et al., 2008). PAMs were maintained in RPMI 1640 medium (Gibco, Carlsbad, CA, United States) supplemented with 10% fetal bovine serum (FBS) (Biological Institute, Israel), 100 U penicillin/ml, and 100 μg streptomycin/ml. HEK293T and MARC-145 cells were grown in Dulbecco’s modified Eagle’s medium (DMEM) (Gibco) supplemented with 10% FBS (Biological Institute). HEK293T cells are not permissive for PRRSV infection, but can be transfected with host cellular genes to study the roles of expressed proteins on PRRSV infection. All cells were cultured, unless otherwise noted, at 37°C with humidity and 5% CO_2_.

Various PRRSV-2 isolates (with GenBank accession number as listed) were studied in this work and included the following: two highly pathogenic PRRSV strains, JXA1 (GenBank: EF112445.1) and SD16 (GenBank: JX087437.1), PRRSV-2 prototype strain VR-2332 (GenBank: EF536003.1), Chinese classical strain CH-1a (GenBank ID: AY032626.1), and two PRRSV-1 isolates GZ11-G1 (GenBank ID: KF001144.1) and P073-3 (only partially sequenced and confirmed as a PRRSV-1 isolate, with no full sequence available). Virus stocks were used to infect various cell lines at 0.1 to 50 multiplicity of infection (MOI). Viral titers were determined in MARC-145 cells by calculating the median tissue culture infective dose (TCID_50_) as previously described (Du et al., 2016).

### Plasmids and Antibodies

To achieve co-expression of two recombinant host receptor-based proteins in HEK293T cells, mammalian expression vector pCAGEN (plasmid #11160, Addgene, Watertown, MA, United States) was modified to serve as a bicistronic expression system by the insertion of the internal ribosome entry site (IRES) sequence amplified from pTRIP-CMV-IRES-Puro into the multiple cloning site of pCAGEN (Li et al., 2015). The cDNAs of MYH9 C-terminal domain (PRA) and full length CD163 or CD163 truncations were inserted into bicistronic pCAGEN (Supplementary Figure S1). All HEK293T cell transfections were conducted using FuGENE-HD Transfection Reagent (Promega, Madison, WI, United States) following the manufacturer’s instructions. Sequencing of new vector constructs was performed to confirm that introduced sequences, junctions, and arrangement of insertions within the parent vector were correct.

Mouse anti-myc tag monoclonal antibodies (Mab), anti-His Mab, anti-HA Mab, and rabbit anti-MYH9 polyclonal antibodies were purchased from Protein Tech (Rosemont, IL, United States). Mouse Mabs against PRRSV N protein (Clone No. 6D10), CD163 and anti-idiotypic antibody recognizing MYH9 (Mab2-5G2) were produced and maintained in house as previously described (Li et al., 2015). Anti-α-tubulin Mab, and anti-Vimentin Mab were purchased from Sigma-Aldrich (St. Louis, MO, United States). Horseradish peroxidase (HRP)-conjugated goat anti-mouse IgG (H + L) and goat anti-rabbit IgG (H + L) secondary antibodies and Alexa Fluor 488-labeled goat anti-mouse IgG and 555-labeled goat anti-rabbit IgG secondary antibodies were purchased from Invitrogen (Carlsbad, CA, United States).

### Co-immunoprecipitation Assay (Co-IP)

Porcine alveolar macrophages were seeded into 10-cm dishes and inoculated with PRRSV-2 strain JXA1 at 0.1 MOI and incubated for 48 h. HEK293T cells were cultured in 10-cm dishes and cell monolayers were transfected with bicistronic expression plasmids encoding both full-length PRA and full-length CD163 or CD163 truncations for 48 h. HEK293T cells stably expressing CD163, CD163 SRCR1-4, or CD163 SRCR5-CT were cultured in 10-cm dishes and cell monolayers were used to generate cell lysates.

Whole-cell lysates of infected or transfected cells were suspended in co-IP buffer [0.5% Triton X-100 (Sigma-Aldrich), 50 mM Tris–HCl (pH 7.4), 150 mM NaCl, 0.2 mM EDTA, 2 mM EGTA, 10% Glycerol] supplemented with 1 × protease inhibitor cocktail (Roche, Basel, Switzerland). Cell lysates were clarified by centrifugation at 15,000 × *g* for 10 min at 4°C.

To reduce non-specific background, cell lysates were precleared with protein G agarose beads (Pierce, Rockford, IL, United States) for 1 h at 4°C. Each supernatant was subjected to immunoprecipitation using appropriate antibodies at 4°C for 12 h. Next, fresh protein G agarose beads were added and the suspensions were incubated for 6 h at 4°C. Beads bound to immune complexes were washed in co-IP buffer four times and were finally suspended in 2 × Laemmli sample buffer to generate samples for SDS–PAGE and Western blot analyses.

### Western Blot Analysis

Cells were lysed using ice-cold NP-40 or RIPA lysis buffer (Beyotime) supplemented with 1 mM PMSF (Beyotime) and mixed with 2 × Laemmli sample buffer for SDS–PAGE. Equal amounts of protein samples were loaded onto 12% SDS–PAGE gels and separated proteins were transferred onto PVDF membranes as described previously (Zhang et al., 2017). Membranes were blocked with 1% BSA in PBS and probed with the indicated antibodies. Specific binding of antibodies to their targets was detected with HRP-conjugated secondary antibodies and visualized using ECL substrate (Beyotime). Chemiluminescence signal acquisition was conducted using a ChemiDoc MP imaging system (Bio-Rad Laboratories, Hercules, CA, United States) and analyzed using Image Lab software (Version 5.1, Bio-Rad Laboratories).

### Membrane Protein Extraction

Porcine alveolar macrophages were seeded into wells of 6-well plates and cytoplasmic and membrane fractions were extracted according to instructions provided with the ProteoExtract Native Membrane Protein Extraction Kit (Calbiochem, San Diego, CA, United States).

For detection of an interaction between SRCR1-4-His and membrane-localized MYH9, PAMs were seeded into 6-well plates and incubated for 3 h. Next, SRCR1-4-His was added to wells then plates were incubated for 2 h at 37°C. Cellular membrane preparation prior to the co-IP assay was conducted as previously described (Xiong et al., 2015) with the following modifications. Briefly, after washing cells with PBS buffer, cells were cross-linked with 3,3′-dithiobis(sulfosuccinimidyl propionate) (DTSSP) (Pierce, Rockford, IL, United States) at a final concentration of 2 mM in PBS for 30 min at 37°C. The reaction was terminated by addition of 50 mM Tris buffer (pH 7.5) with incubation at 37°C for 15 min. Cytoplasmic and membrane fractions were separated using the extraction kit as mentioned above to conduct the co-IP assay to verify the MYH9 in which fractions interacted with SRCR1-4-His.

### Expression of Recombinant SRCR1-4 Protein

Protein expression and purification were conducted as previously described (Chen et al., 2018) with the following modifications. Briefly, the sequence encoding the SRCR1-4 region was cloned from PAMs cDNA and inserted into the pET-21a vector. After transforming *E. coli* BL21 (DE3) (TransGen Biotech, Beijing, China) with vector containing the inserted SRCR1-4, bacteria were cultured in LB medium. After IPTG (1.0 mM) induction for 6 h at 37°C, bacterial cells were collected and resuspended in cell lysis buffer [50 mM Tris–HCl (pH 7.5), 150 mM NaCl, 1 mM EDTA, 1 mM AEBSF (4-benzenesulfonyl fluoride hydrochloride), 5% glycerol] and sonicated. Inclusion bodies containing SRCR1-4-His protein were washed with Buffer A (50 mM Tris–HCl (pH 8.0), 300 mM NaCl, 10 mM EDTA, 10 mM DTT, 0.5% Triton X-100) then dissolved in Buffer B (50 mM Tris–HCl (pH 8.0), 100 mM NaCl, 5 mM EDTA, 5 mM DTT, 10% glycerol, 8 M urea) and incubated for 6-8 h at 4°C. SRCR1-4-His protein was purified using Ni^+^ affinity chromatography (Roche) and then eluted with Buffer B containing 200 mM imidazole followed by dialysis in Buffer C [100 mM Tris–HCl (pH 8.0), 400 mM L-arginine monohydrochloride, 2 mM EDTA, 5 mM L-glutathione (reduced form), and 0.5 mM oxidized L-glutathione]. Purity of SRCR1-4-His protein was analyzed using SDS–PAGE and Western blot as described above.

### Enzyme-Linked Immunosorbent Assays (ELISAs)

Recombinant PRA proteins were expressed and purified as previously described (Li et al., 2018). Indirect ELISAs were conducted to detect the interaction between recombinant SRCR1-4 and PRA proteins. Briefly, 96-well polystyrene microtiter plates (Corning, NY, United States) were coated with recombinant proteins at 400 ng/well in PBS buffer (pH 8.0) overnight at 4°C followed by blocking with 1% BSA in PBS-T buffer (PBS containing 0.5% Tween 20, Sigma-Aldrich) before use. 1, 0.1, 0.01, and 0.001 μg of the interaction partner were added to wells followed by incubation for 1 h at 37°C. After three washes with PBS-T buffer, the presence of an interaction was detected by the binding of anti-His or Mab2-5G2 antibodies followed by development with tetramethylbenzidine substrate (Beyotime) to form a visible product. The values of absorbance at 450 nm were evaluated using a Victor^TM^ X5 Multilabel Plate Reader (PerkinElmer, Waltham, MA, United States). Home-made recombinant swine hepatitis E virus ORF2 protein (239) or PBS were used as the negative or blank controls.

### Far-Western Blot Assay

To further examine the interaction between SRCR1-4 and PRA, far-Western blots were conducted. Briefly, purified SRCR1-4-His or PRA were resolved by SDS–PAGE and separately transferred onto PVDF membranes. After blocking unbound sites, membranes were probed with either PRA or SRCR1-4-His dissolved in PBS buffer for 2 h at 37°C. A PRA-SRCR1-4 interaction was detected with Mab2-5G2 or anti-His Mab and visualized by the addition of HPR-labeled goat anti-mouse IgG (H + L) followed by ECL substrate.

### Confocal Microscopy

The assay was performed as previously described (Zhao et al., 2018). PAMs were seeded onto glass coverslips, each with a diameter of 14 mm, and cells on coverslips were incubated with SRCR1-4-His for 2 h at 37°C. After washing with PBS, cells were fixed with 4% formaldehyde solution for 15 min at room temperature then blocked using 1% BSA in PBS for 1 h at room temperature. Next, cells were probed with mouse anti-His Mab and rabbit anti-MYH9 polyclonal antibodies in 1% BSA for 1 h at 37°C. After washing with PBS containing 0.5% Triton X-100 (Sigma-Aldrich), secondary antibodies Alexa Fluor 488-labeled goat anti-mouse IgG or Alexa Fluor 555-labeled goat anti-rabbit IgG were incubated with the cells for another 1 h at 37°C. Next, specimens were mounted onto slides using ProLong^®^ Gold Antifade Reagent containing 4′,6-diamidino-2-phenylindole (DAPI) (Thermo Fisher Scientific, Waltham, MA, United States). Image acquisition was conducted using an LSM 800 ZEISS Confocal Laser Scanning Microscope with Airyscan (Carl Zeiss AG, Oberkochen, Germany). Using the Manders’ overlap coefficient, colocalization was analyzed between SRCR1-4 and MYH9 using ImageJ software.

### Establishment of HEK293T Cell Lines That Stably Express CD163 or Truncated CD163

HEK293T cells with stable expression of CD163 or CD163 truncations were generated by lentiviral transduction followed by puromycin selection. Briefly, lentiviral particles bearing CD163, CD163 SRCR1-4, or CD163 SRCR5-CT were constructed by inserting corresponding cDNA inserts into pTRIP-CMV-IRES-Puro plasmid and co-transfecting these recombinant plasmids into HEK293T cells along with helper packaging plasmids psPAX2 and pMD2.G. Supernatants containing lentiviral particles were harvested and transduced to establish stably expressing HEK293T^CD163^, HEK293T^SRCR1–4^, and HEK293T^SRCR5–CT^ cells followed by puromycin selection (30 μg/ml, Merck, United States). Subcloning of surviving cells was performed using limiting dilution in 96-well plates.

### Immunofluorescence Assays (IFA)

HEK293T cells stably expressing CD163 and truncated proteins were grown on coverslips in 24-well plates then fixed with 4% paraformaldehyde (Sigma-Aldrich), permeabilized with PBS containing 0.5% Triton X-100 (Sigma-Aldrich), and blocked with PBS containing 1% BSA (Sigma-Aldrich). Cells were stained with indicated antibodies at 37°C for 1 h. Specific binding between antibodies and targets was detected with Alexa Fluor 488-labeled goat anti-mouse IgG. Coverslips were mounted onto slides using ProLong^®^ Gold Antifade Reagent containing 4′,6-diamidino-2- phenylindole (DAPI) (Thermo Fisher Scientific) and observed using fluorescence microscopy (EVOS FL, Thermo Fisher Scientific).

### Analysis of PRRSV Attachment, Internalization, Disassembly, and Infection

HEK293T^CD163^, HEK293T^SRCR5–CT^, and HEK293T^SRCR1–4^ cells were seeded onto coverslips in 24-well plates and inoculated with PRRSV strain JXA1 at a MOI of 50. Detection of PRRSV attachment, internalization, disassembly, and infection in each cell line were performed via IFA as previously described (Delrue et al., 2010; Du et al., 2017; Li et al., 2017). Briefly, in order to assess the virion attachment, viruses were incubated with cells at 4°C for 1 h, and fixed with 4% formaldehyde solution. To analyze the internalized virion, cells with attached viruses (as described above) were transferred to 37°C and cultured for an another 1 h to allow internalization to occur followed by proteinase K (Sigma) treatment for 45 min at 4°C to remove non-internalized virions. The protease reaction was terminated by the addition of protease inhibitor cocktail (Roche) and incubation at 4°C for 2 min. For the virion disassembly and infection assay, the cells with internalized viruses (as described above) were incubated at 37°C for an additional 5and 24 h, followed by fixed with 4% formaldehyde solution and permeabilized with 0.1% Triton X-100 as described previously. The virion was stained with 6D10 and secondary Alexa Fluor 488 Goat anti mouse IgG antibodies. Cells were imaged with an LSM800 ZEISS Confocal Laser Scanning Microscopy with Airyscan (Zeiss).

Meanwhile, cells were seeded into 12-well plates and inoculated with JXA1 at a MOI of 50 (and in parallel experiments with JXA1 at a MOI of 10) for detection of virion attachment and internalization via Western blot. Cells were inoculated with virus at 4°C, incubated for 1 h, then harvested for virion attachment analysis. For the PRRSV internalization assay, cells with attached virus (as described above) were transferred to 37°C and cultured for an additional 1 h to allow internalization to occur followed by proteinase K (Sigma) treatment for 45 min at 4°C to remove non-internalized virions. The protease reaction was terminated by the addition of protease inhibitor cocktail (Roche) and incubation at 4°C for 2 min. Next, cells were harvested for subsequent Western blot analysis.

### RNA Isolation and Quantitative Real-Time PCR (qPCR)

All cell samples were harvested for qPCR analysis at 24 h postinfection (hpi) unless otherwise noted, then total RNA was extracted from the various types of cells using TRizol reagent (Thermo Fisher Scientific) in accordance with the manufacturer’s instructions. Reverse transcription and qPCR were conducted using a PrimeScript RT reagent Kit (TaKaRa, Dalian, China). GAPDH transcripts were also amplified and used to normalize total RNA input. As a recent study has demonstrated a dose-dependent association between CD163 mRNA expression level and virus infection (Wang et al., 2018), here SRCR1-4 and SRCR5-CT transcripts were amplified and normalized using total RNA input to quantify virus infection of HEK293T^CD163^, HEK293T^SRCR1–4^, and HEK293T^SRCR5–CT^ cells. The primers and corresponding sequence used for qPCR amplification were listed in Table 1. Relative quantities of target genes were calculated using the 2^–Δ^ ^Δ^ ^Ct^ method.

**TABLE 1:** Sequence of the primers of qPCR.

**Primer**	**Sequence**	**(5′-3′)**
PRRSV ORF7	Forward	ATGCCAAATAACAACGGCAAGCAGC
	Reverse	TCATGCTGAGGGTGATGCTGTG
GAPDH	Forward	CCTTCCGTGTCCCTACTGCCAAC
	Reverse	GACGCCTGCTTCACCACCTTCT
SRCR1-4	Forward	CAAAGCCACTGGATGGGCTA
	Reverse	CCAGCATCCTGTTGGTGAGT
SRCR5-CT	Forward	GAGTTGCCCTTTCTATCCCG
	Reverse	GAGCAGTGACGGAACAATCT

### PRRSV Inhibition Assay

Porcine alveolar macrophages were seeded into 24-well plates (5 × 10^5^ cells/well). Next, recombinant SRCR1-4-His proteins at 0, 1, 2.5, 5, and 10 μM were added to each well then plates were incubated for 2 h at 37°C. The unbound proteins were removed by washing cells with PBS. PRRSV-1 and PRRSV-2 viral isolates were used to inoculate PAMs at 0.1 MOI for 1 h at 37°C followed by washing with PBS to remove unbound virions. At 24 h after virus innoculation, PAMs and cell culture supernatants were harvested for qPCR, Western blot analysis, and IFA to evaluate virus replication.

### Cell Viability Assay

Cytotoxicity of purified CD163 SRCR1-4-His toward PAMs was evaluated using a Cell Counting Kit-8 (CCK-8) (Beyotime) according to the manufacturer’s instructions and described previously (Li et al., 2018). Briefly, PAMs were seeded into 96-well plates (1 × 10^5^ cells/well) and incubated with SRCR1-4-His protein at various concentrations at 37°C for 24 h. Next, CCK-8 reagent was added (10 μL/well) followed by incubation for another 2 h. Viable cells were determined from absorbance readings (450 nm) using a Victor^TM^ X5 Multilabel Plate Reader (PerkinElmer).

### Statistical Analysis

All experiments were conducted independently at least in triplicate. The ELISA results in Figure 3, cell viability assay results in Figure 9, and qPCR results in Figures 7, 9, 10 were analyzed using GraphPad Prism version 5.0 (GraphPad Software, San Diego, CA, United States). Statistical significance was determined by student’s *t*-test for comparison of two groups or by one-way analysis of variance (ANOVA) for testing of more than two groups. *P* < 0.05 was considered statistically significant.

## Results

### Interaction Between CD163 and MYH9 in PAMs

**FIGURE 1:**
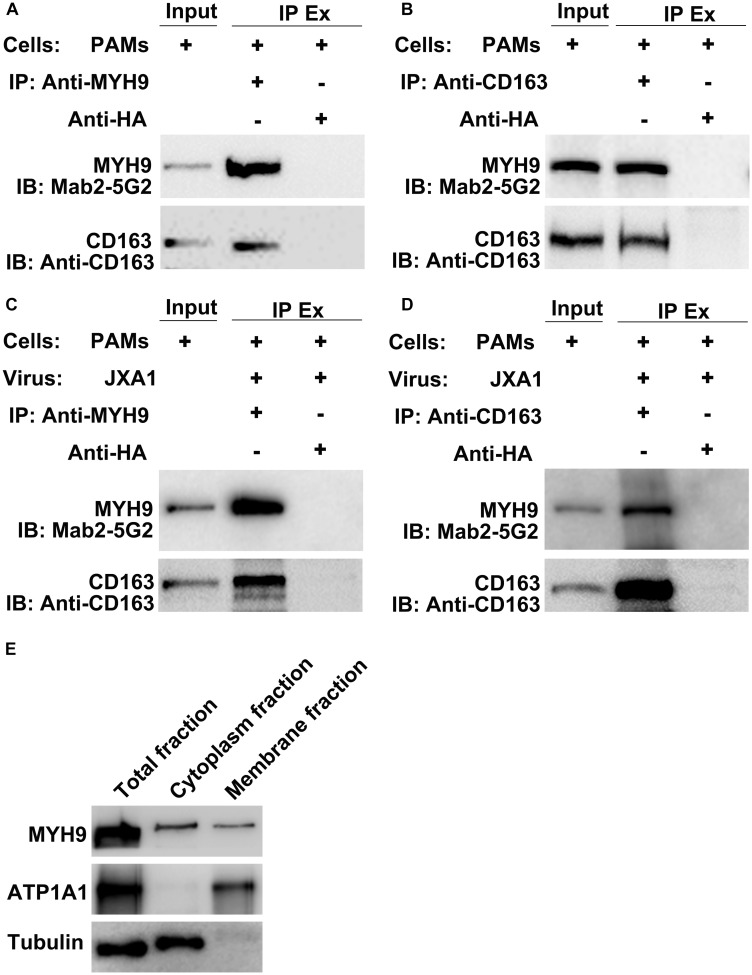
CD163 interacts with MYH9 in PAMs regardless of PRRSV infection status. Input means initial extract, and IP Ex represents immunoprecipitated extracts. Lysed PAMs were immunoprecipitated with anti-MYH9 pAb to detect CD163 **(A)** and with anti-CD163 Mab to detect MYH9 **(B)**. PAMs were infected with PRRSV strain JXA1 at 0.1 MOI for 48 hpi, lysed, and immunoprecipitated with anti-MYH9 pAb to detect CD163 **(C)** and with anti-CD163 Mab to detect MYH9 **(D)**. **(E)** Membrane-associated MYH9 in PAMs was extracted using a Membrane Protein Extraction Kit and anti-MYH9 pAb was used to detect MYH9 distribution in whole cell lysates and cytoplasmic and membrane fractions. Antibodies against ATP1A1 and α-tubulin were used to normalize overall membrane or cytoplasm protein levels to allow comparisons of proteins for equivalent numbers of cells.

CD163 has been demonstrated to be an essential receptor for PRRSV infection both *in vitro* and *in vivo* (Calvert et al., 2007; Guo et al., 2014; Burkard et al., 2017, 2018; Yang et al., 2018). Our previous studies had shown that in addition to CD163, MYH9 is also required for PRRSV infection of COS7 cells *in vitro* (Gao et al., 2016; Guo et al., 2016). However, it is still not known whether the interaction between CD163 and MYH9 is direct or indirect. Thus, co-IP was conducted using PAMs extracts to examine the interaction between CD163 and MYH9 in the presence or absence of PRRSV infection. The results showed that both proteins immunoprecipitated (IP) together either from uninfected PAMs (Figures 1A,B) or PAMs infected with PRRSV-2 JXA1 strain (Figures 1C,D), thus demonstrating that CD163 and MYH9 interacted in PAMs regardless of PRRSV infection status. In addition, an assessment of the cellular distribution of MYH9 within PAMs demonstrated that although MYH9 was mainly present within the cytoplasm, a significant amount of MYH9 was also associated with the plasma membrane (Figure 1E). Indeed, these results align with the known intracellular distribution of MYH9 for other cell types (Olden et al., 1976; Nebl et al., 2002; Xiong et al., 2015).

### The CD163 SRCR1-4 Domain Directly Interacts With the MYH9 C-Terminal Region

**FIGURE 2:**
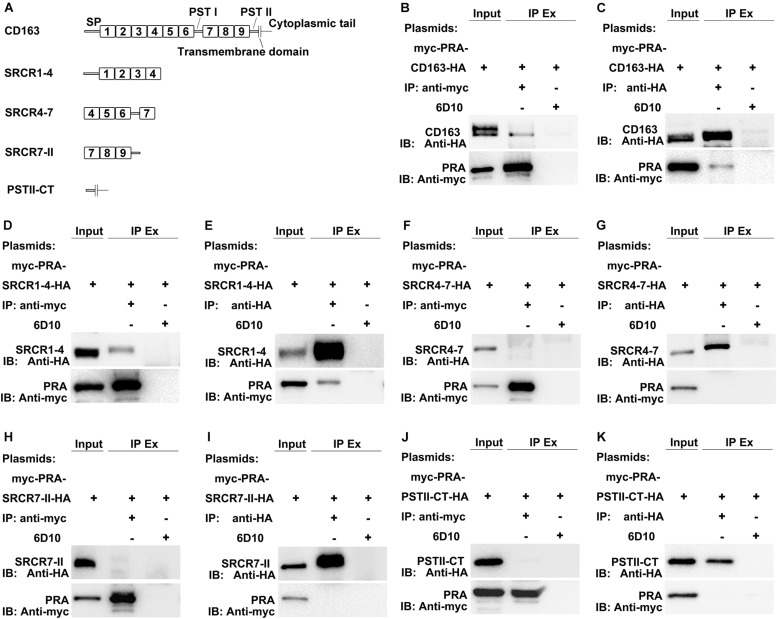
The SRCR1-4 domain of CD163 interacts with PRA, the C-terminal region of MYH9. **(A)** A schematic diagram of full-length and truncated CD163 proteins. HEK293T cells transfected with bicistronic plasmids co-expressing myc-PRA and CD163-HA or truncated CD163-HA were harvested after 48 h transfection. Cell lysates were immunoprecipitated with anti-myc Mab **(B,D,F,H,J)** or anti-HA Mab **(C,E,G,I,K)** and both initial extracts and immunoprecipitated extracts (IP Ex) were analyzed by Western blotting using anti-myc Mab or anti-HA Mab. A Mab (6D10) specific for PRRSV N protein (made in house) was used as an isotype control for co-IP.

MYH9 shares a high level of amino acid sequence similarity with MYH10, with the exception of sequences within the non-helical tail region of the MYH9 C-terminal region (designated PRA) (Li et al., 2018). Due to the fact that the PRA region appears to be crucial for PRRSV infection (Gao et al., 2016), PRA was subsequently analyzed to more precisely define the CD163-interacting region. CD163 is composed of a signal peptide, nine SRCR domains, two PST-rich regions, a transmembrane domain, and a cytoplasmic tail (Ma and Adelstein, 2014; Sun et al., 2014). To identify the CD163 region that binds to PRA, recombinant constructs containing the PRA protein coding sequence and protein coding sequences of either full-length CD163 or four CD163 truncations, designated SRCR1-4, SRCR4-7, SRCR7-II, and PSTII-CT, were cloned into a bicistronic expression vector (Figure 2A) then transfected into HEK293T cells. Co-IP was conducted using anti-myc Mab or anti-HA Mab to detect reciprocal interactions between PRA and full-length or truncated CD163 proteins. As expected, PRA co-precipitated with full-length CD163, and vice versa (Figures 2B,C). Furthermore, co-precipitation of PRA and SRCR1-4 was observed (Figure 2D) and consistent results were obtained from reciprocal experiments (Figure 2E) that collectively demonstrate that PRA interacted with the CD163 SRCR1-4 domain. By contrast, no such co-precipitation was detected when SRCR4-7 (Figures 2F,G), SRCR7-II (Figures 2H,I), or PSTII-CT domain (Figures 2J,K) were expressed with PRA in HEK293T cells.

**FIGURE 3:**
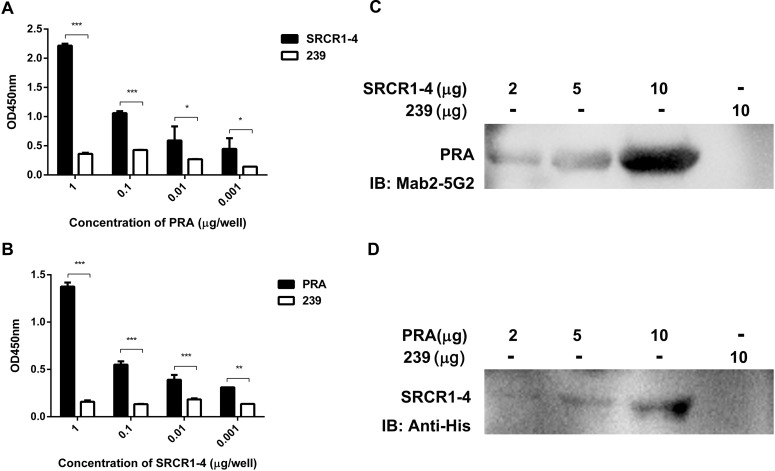
Prokaryotic expressed SRCR1-4 protein directly interacts with PRA. The interaction between prokaryotic expressed recombinant SRCR1-4 protein and PRA was examined using indirect ELISA **(A,B)** and far Western blot analyses **(C,D)**. **(A,B)** Recombinant SRCR1-4-His or PRA protein was used to coat ELISA plates then was allowed to interact with added PRA or SRCR1-4-His at indicated concentrations. ^*^P < 0.05; ^∗∗^*P* < 0.01; and ^∗∗∗^*P* < 0.001. **(C,D)** Purified SRCR1-4-His and PRA were each separated by SDS–PAGE and transferred to PVDF membranes. After blocking, membranes were probed with PRA or SRCR1-4-His at indicated concentrations and designated antibodies were used to detect the interaction. Protein 239 (recombinant swine hepatitis E virus ORF2 protein) was used as an irrelevant protein control.

Although a potential interaction between CD163 SRCR1-4 domain and MYH9 PRA was revealed via co-IP assays, it is still unclear whether this is a direct interaction or is indirectly mediated via other adaptor proteins that bridge the connection, especially since MYH9 has been demonstrated to have other interaction partners (Lin et al., 2012). To learn more about this interaction, recombinant SRCR1-4 protein was expressed in *E. coli*, purified (Supplementary Figure S2), then assessed for direct binding to recombinant porcine PRA protein as previously described (Calvert et al., 2007) via indirect ELISA and far-Western blot analyses. The results showed that SRCR1-4 directly bound to PRA in a dose-dependent manner (Figures 3A,C), and vice versa (Figures 3B,D). Moreover, the interaction between SRCR1-4 and PRA was specific, since SRCR1-4 or PRA did not bind to the irrelevant control protein, truncated recombinant swine hepatitis E virus ORF2 protein (designated 239). Thus, the results here demonstrate that the SRCR1-4 domain directly interacted with MYH9 C-terminal region protein PRA.

### Recombinant SRCR1-4 Directly Interacts With Membrane MYH9

**FIGURE 4:**
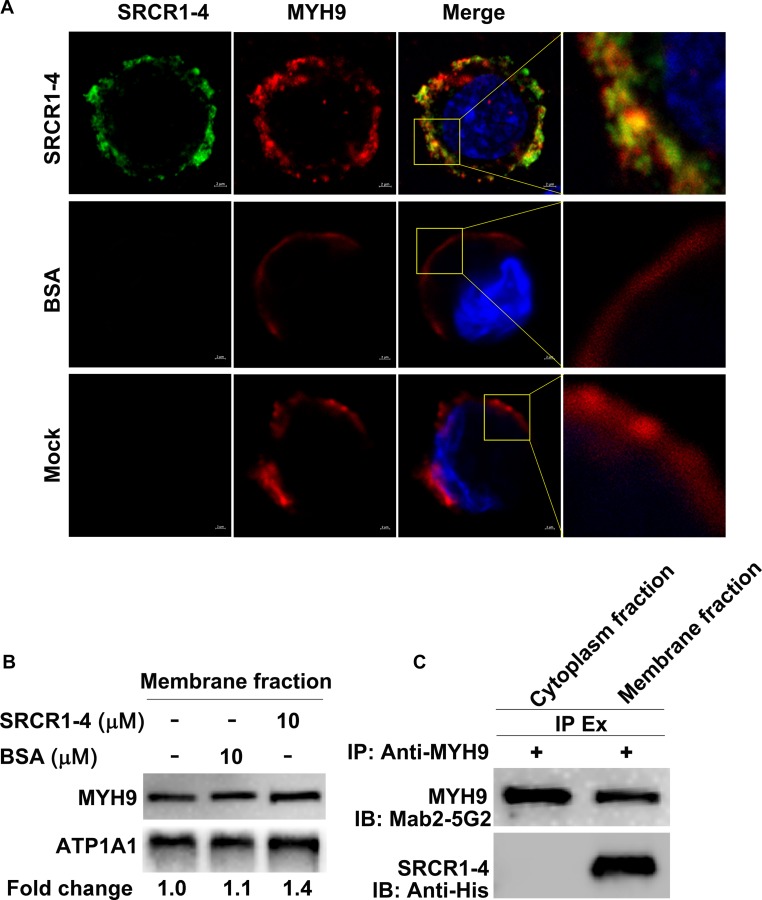
Recombinant SRCR1-4 protein directly interacts with MYH9 in PAMs. **(A)** PAMs were incubated with SRCR1-4 or the same quantity of BSA. Cells were subjected to immunofluorescence staining of MYH9 (red) and SRCR1-4 (green) and cellular nuclei were counterstained with DAPI (blue). Co-localization of MYH9 and SRCR1-4 was visualized by confocal microscopy. **(B)** The membrane fraction of SRCR1-4 or BSA-preincubated PAMs was extracted using the Membrane Protein Extraction Kit followed by Western blot analysis to detect MYH9 expression. **(C)** PAMs were pre-incubated with SRCR1-4 or BSA. Cytoplasmic and membrane fractions were separated using a Membrane Protein Extraction Kit and then each fraction was immunoprecipitated with anti-MYH9 pAb to detect the interaction between MYH9 and recombinant SRCR1-4 protein.

Since prokaryotic expressed protein SRCR1-4 directly interacted with prokaryotic expressed PRA (Figure 3), we next addressed whether SRCR1-4 incubated with PAMs directly interacted with MYH9 located on the plasma membrane. Using non-permeabilized cells, both recombinant SRCR1-4 and endogenous MYH9 were shown to be distributed on the membrane and possessed a Manders’ overlap coefficient of 0.91 (Figure 4A), indicating that recombinant SRCR1-4 co-localized with membrane-distributed MYH9.

Surprisingly, when recombinant SRCR1-4 was incubated with PAMs, more MYH9 molecules were found to be distributed on the plasma membrane as compared with the mock control (Figure 4A), which was further confirmed by evaluation of membrane MYH9 expression via Western blot analysis (Figure 4B). Furthermore, results of the co-IP assay demonstrated that recombinant SRCR1-4 could only co-immunoprecipitate with plasma membrane-associated MYH9, but not with MYH9 localized within the cytoplasm of PAMs (Figure 4C). Thus, recombinant SRCR1-4 directly interacted with MYH9 associated with the plasma membrane of PAMs.

### The Crucial Role of CD163 SRCR1-4 in Mediating PRRSV Internalization

**FIGURE 5:**
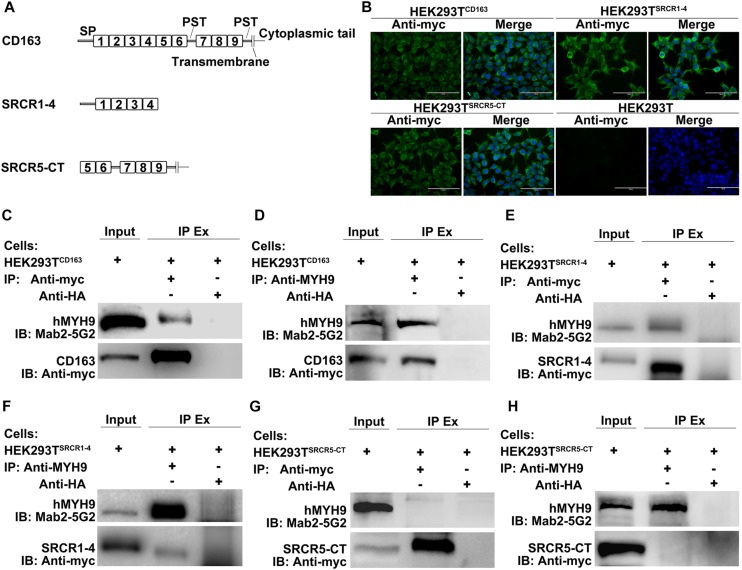
SRCR1-4 interacts with human MYH9 (hMYH9) when stably expressed in HEK293T^SRCR1– 4^ cells. **(A)** A schematic diagram of full-length and truncated CD163 proteins. **(B)** HEK293T cells were transduced with lentivirus expressing CD163, SRCR1-4, and SRCR5-CT then puromycin-resistant cells were selected and subcloned. Expression of CD163, SRCR1-4, and SRCR5-CT by cell lines was detected by immunofluorescence using anti-myc Mab. Original un-transduced HEK293T cells served as a negative control. Cells were lysed and lysates were immunoprecipitated with anti-myc **(C,E,G)** or anti-MYH9 antibody **(D,F,H)**. Whole-cell lysates and immunoprecipitated antibody-antigen complexes were subjected to Western blot analysis and detected using Mab2-5G2 and anti-myc antibodies.

As the data depicted above demonstrated that a direct interaction occurred between SRCR1-4 and MYH9, we next worked to reveal the biological function of this interaction. Since HEK293T cells support PRRSV replication after introduction of porcine CD163 (Van Gorp et al., 2010), HEK293T cell lines expressing CD163 and CD163 truncations were used to address whether their direct interaction would influence the PRRSV replication cycle. Specifically, cell lines designated HEK293T^CD163^, HEK293T^SRCR1–4^, and HEK293T^SRCR5–CT^, were generated via transduction of HEK293T cells with lentivirus encoding porcine full-length CD163, SRCR1-4, or SRCR5-CT, respectively (Figure 5A). First, expression of CD163, SRCR1-4, or SRCR5-CT proteins in each cell line was confirmed by immunofluorescence assay (IFA) (Figure 5B). Meanwhile, the co-IP assay further verified that both porcine full-length CD163 (Figure 5C) and SRCR1-4 (Figure 5E) could bind to human MYH9 (hMYH9), with consistent results obtained from reciprocal experiments (Figures 5D,F). In contrast, no SRCR5-CT bound to hMYH9 (Figures 5G,H).

**FIGURE 6:**
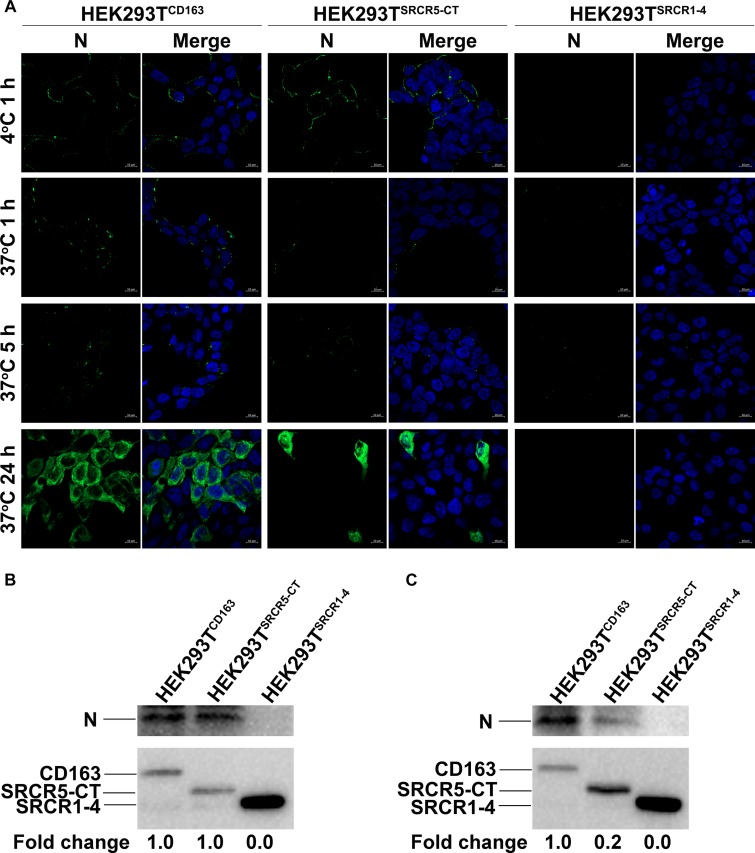
The SRCR1-4 domain is crucial for PRRSV internalization. **(A)** HEK293T^CD163^, HEK293T^SRCR5–CT^, and HEK293T^SRCR1– 4^ cell lines were inoculated with PRRSV strain JXA1 at 50 MOI. After incubation at 4°C for 1 h to allow virus attachment, the temperature was shifted to 37°C for 1, 5, and 24 h to trigger virus internalization, disassembly, and infection, respectively. Various sequential stages of the viral replication cycle were measured by immunofluorescence staining of virus using 6D10 (antibody against PRRSV N protein) and visualized under confocal microscopy. **(B)** After virus attachment for 1 h, cells were lysed and subjected to Western blot analysis using 6D10 and anti-myc antibodies. Values are normalized to HEK293T^CD163^ cells infected with PRRSV with the fold change in relative expression as indicated. **(C)** Cells were lysed after virus internalization then were subjected to Western blotting as described in panel **(B)**.

We next analyzed the PRRSV replication cycle in HEK293T^CD163^, HEK293T^SRCR1–4^, and HEK293T^SRCR5–CT^ cell lines. As shown in Figure 6A, PRRSV attachment, internalization, disassembly, and infection stages were analyzed by confocal microscopy. However, after incubation with virus at 4°C, attachment of virus particles was only observed for HEK293T^CD163^ and HEK293T^SRCR5–CT^ cells, but not for HEK293T^SRCR1–4^ cells, (Figure 6A, first top panel), indicating no SRCR1-4 involvement during the PRRSV attachment stage. Next, a temperature shift to 37°C triggered internalization of viral particles, with an apparent decrease in PRRSV internalization observed in HEK293T^SRCR5–CT^ cells compared with HEK293T^CD163^ cells (Figure 6A, second top panel). Decreased internalization in these cell lines further resulted in significantly impaired PRRSV disassembly and infection efficiency (Figure 6A, first and second bottom panels, respectively), indicating that SRCR1-4 may participate in the PRRSV internalization process.

**FIGURE 7:**
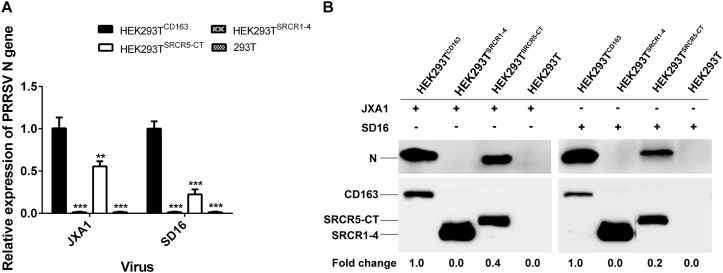
SRCR1-4 domain expression affects viral replication efficiency. **(A)** The HEK293T^CD163^, HEK293T^SRCR1– 4^, and HEK293T^SRCR5–CT^ cell lines were infected with PRRSV strain JXA1 or SD16 at a MOI of 0.1, with HEK293T cells serving as the negative control. The RNA level of PRRSV-N expression was determined by qPCR. Values were normalized to HEK293T^CD163^ infected with PRRSV. ^∗∗^*P* < 0.01 and ^∗∗∗^*P* < 0.001. **(B)** After infection with JXA1 or SD16 for 24 h, cells were lysed and subjected to Western blot analysis using 6D10 and anti-myc antibodies. Values are normalized to HEK293T^CD163^ cells infected with PRRSV with fold difference in relative expression as indicated.

Similar results were found regarding PRRSV-N protein expression by cell lines during PRRSV attachment and internalization stages, as shown using Western blot analysis, with comparable attachment of viral particles observed for HEK293T^CD163^ and HEK293T^SRCR5–CT^ cells (Figure 6B). Moreover, contrary to observations of HEK293T^CD163^ cells, significantly diminished internalization of virus particles was observed by HEK293T^SRCR5–CT^ cells (Figure 6C). Consequently, in HEK293T^SRCR5–CT^ the lack of the SRCR1-4 domain significantly reduced PRRSV replication, as assessed from reduced PRRSV-N expression at both RNA and protein levels (Figures 7A,B). Conversely, HEK293T^SRCR1–4^ cells also did not support PRRSV replication, but this result was due to the lack of initial viral particle attachment. Together, these findings demonstrate different roles of SRCR1-4 and SRCR5-CT domains in the PRRSV replication cycle; SRCR1-4 played a critical role in PRRSV internalization, while SRCR5-CT participated in virus attachment, with both domains contributing to viral replication.

### CD163 SRCR1-4 Mediates PRRSV Internalization via Direct Interaction With MYH9 in PAMs

The data as depicted in Figures 6A,C show that the PRRSV internalization step was severely compromised in cells with CD163 SRCR1-4 deficiency. In conjunction with our findings from Figure 4C, this indicated that recombinantly expressed SRCR1-4 directly interacted with MYH9 in PAMs. Therefore, we hypothesized that the addition of recombinant SRCR1-4 to PAMs would interfere with PRRSV internalization and sequential infection via competitive inhibition for MYH9 binding and thus would interrupt the endogenous CD163 and MYH9 interaction.

**FIGURE 8:**
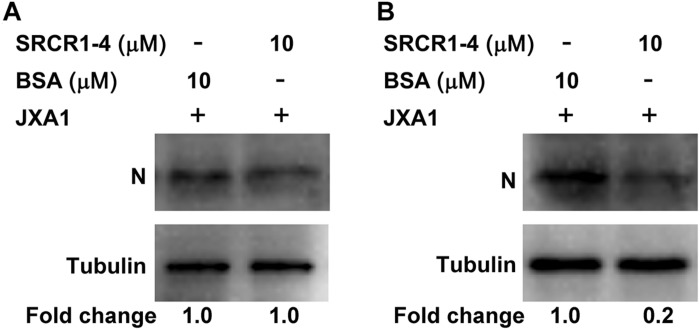
Recombinant SRCR1-4 protein significantly inhibits PRRSV internalization by PAMs. **(A)** Recombinant CD163 SRCR1-4-His protein or BSA were incubated with PAMs then cells were inoculated with JXA1 at 10 MOI for 1 h at 4°C. Cells were lysed and subjected to Western blot analysis using 6D10 and anti-α tubulin antibodies. Values are normalized to those of HEK293T^CD163^ cells treated with BSA, with the fold change in relative expression as indicated. **(B)** Recombinant CD163 SRCR1-4-His protein or BSA was incubated with PAMs then cells were inoculated with JXA1 at 10 MOI for 1 h at 4°C and cultured for 1 h at 37°C. Cells were lysed after virus internalization then were subjected to Western blotting as described in panel **(A)**.

**FIGURE 9:**
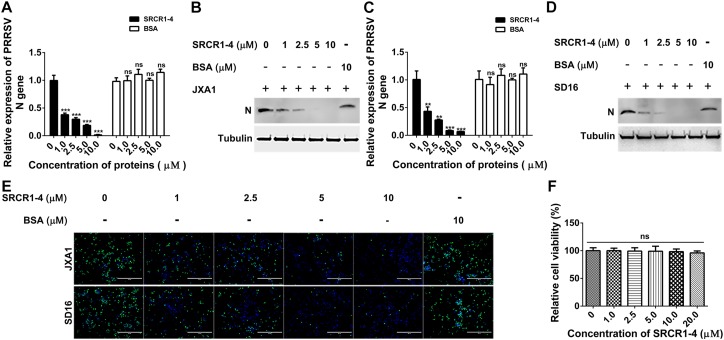
Recombinant SRCR1-4 protein blocks infection of PAMs by highly pathogenic PRRSV (HP-PRRSV). **(A,B)** Recombinant CD163 SRCR1-4-His protein or BSA at the indicated concentration was incubated with PAMs. Next, JXA1 at 0.1 MOI was added to wells and plates were incubated for 24 h. PRRSV-N RNA and protein expression levels were measured using qPCR and Western blotting. Values are normalized to untreated PAMs infected with JXA1. **(C,D)** Pre-treatment with proteins, PRRSV inoculation of PAMs, and subsequent qPCR and Western blot assays were performed as described in A and B for cells inoculated with SD16. **(E)** Pre-treatment of PAMs with proteins followed by addition of PRRSV was performed as described in panels **(A,B)**. Cells were fixed and permeabilized to measure virus replication using immunofluorescence staining of virus using 6D10. **(F)** Recombinant CD163 SRCR1-4-His protein at the indicated concentration was incubated with PAMs and its cytotoxicity for PAMs was measured using a CCK-8 kit. Values were normalized to untreated PAMs. ^∗∗∗^*P* < 0.001; ns, not significant.

To test this hypothesis, attachment, and internalization of viral particles in PAMs infected with JXA1 were evaluated by measurement of PRRSV-N protein production as an indirect measure of virus replication. As expected, PRRSV attachment was unaffected, but internalization was significantly decreased by the addition of exogenous SRCR1-4 to PAMs (Figures 8A,B), with PRRSV replication consequently and markedly diminished by the addition of recombinant SRCR1-4 protein in a dose-dependent manner (Figures 9A,B). Substitution of BSA for SRCR1-4 in a parallel experiment resulted in no effect observed on PRRSV replication cycle, demonstrating the specificity of the effect.

**FIGURE 10:**
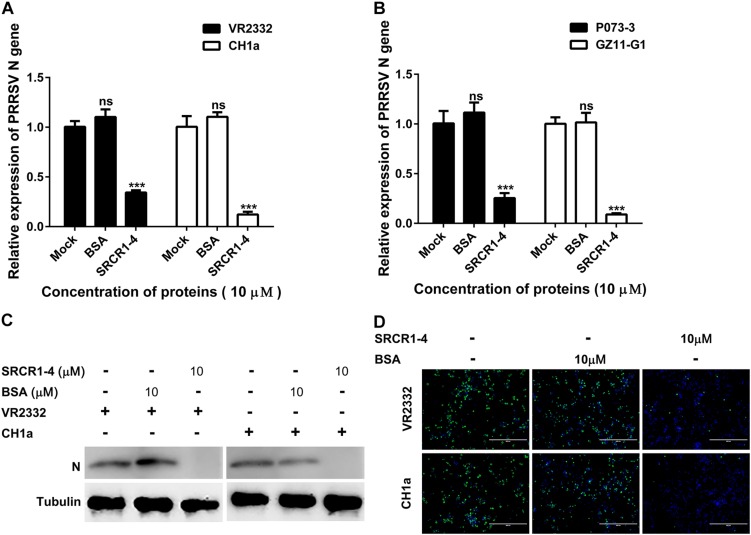
Recombinant SRCR1-4 protein blocks PAMs infection by classical PRRSV-2 and PRRSV-1 strains. **(A,B)** Recombinant CD163 SRCR1-4-His protein (10 μM) or BSA was incubated with PAMs followed by addition to cells of indicated PRRSV strains at 0.1 MOI for 24 h. The RNA level of PRRSV-N expression was detected by qPCR. Values are normalized to values for untreated PAMs infected with PRRSV. ^∗∗∗^*P* < 0.001; ns, not significant. **(C,D)** Pre-treatment of protein and inoculation of PAMs with PRRSV were performed as described in panel **(A)**. Virus replication was measured via Western blotting and immunofluorescence staining with 6D10.

We next tested whether SRCR1-4 exhibited similar inhibition of viral infection of PAMs using heterogeneous PRRSV isolates (including both PRRSV-1 and PRRSV-2). Similar to results for PRRSV strain JXA1, recombinant SRCR1-4 significantly blocked replication of PRRSV-2 (SD16, VR2332, and CH1a) and PRRSV-1 (P073-3 and GZ11-G1) strains, as evidenced by decreased PRRSV N gene RNA levels (Figures 9C, 10A,B), N protein levels (Figures 9D, 10C), and virus infection of PAMs (Figures 9E, 10D). Meanwhile, SRCR1-4 protein exhibited no cytotoxicity for PAMs when in concentrations below 20 μM (Figure 9F). Together, these findings suggest that CD163 SRCR1-4 specifically played a critical role in PRRSV internalization through its direct interaction with MYH9. Notably, the addition of exogenous SRCR1-4 could block this interaction and therefore blocked diverse PRRSV strains infection.

## Discussion

Numerous studies have demonstrated that CD163 is an indispensable cellular receptor for PRRSV infection (Calvert et al., 2007; Whitworth et al., 2016; Burkard et al., 2017; Yang et al., 2018). As a type I membrane protein, the extracellular portion of CD163 is anchored to the cell surface by a single transmembrane region with a short cytoplasmic tail. Of the nine SRCR domains within the extracellular region (SRCR1-9), the SRCR5 domain is particularly crucial and indispensable for successful PRRSV infection (Van Gorp et al., 2010; Wells et al., 2017). Meanwhile, the specific residue at position 561 within the long loop region of the SRCR5 domain has been identified as a key interaction site for PRRSV infection and virion attachment (Ma et al., 2017). However, replacement of the SRCR5 domain of porcine CD163 with the SRCR5 domain of the human CD163-like homolog (CD163Li) only confers resistance to PRRSV-1, but not to PRRSV-2 (Wells et al., 2017). Conversely, COS7 cells with stable CD163 expression are unable to support PRRSV replication unless MYH9 is co-expressed with CD163 (Gao et al., 2016). Above findings suggest that other domains of CD163 besides SRCR5, or even other receptors, are probably involved in PRRSV infection.

MYH9, also known as non-muscle myosin II A heavy chain, was originally identified as a motor protein involved in cell migration, adhesion, and morphogenesis (Ma and Adelstein, 2014). Moreover, MYH9 has been reported to act as a necessary cellular factor for infection with viruses such as herpes simplex virus-1 (HSV-1), thrombocytopenia syndrome virus (SFTSV), Epstein-Barr virus (EBV), and PRRSV (Arii et al., 2010; Sun et al., 2014; Xiong et al., 2015; Guo et al., 2016). As our previous reports had demonstrated, the MYH9 C-terminal domain (PRA) interacted with PRRSV GP5 (Gao et al., 2016; Li et al., 2018). However, it appeared that interruption of the GP5-MYH9 interaction did not inhibit virion binding to cell surfaces, but instead markedly interfered with PRRSV internalization by permissive cells (unpublished data). This result indicates that MYH9 probably does not perform its function as a binding receptor for PRRSV attachment, but acts as a co-receptor for the triggering of PRRSV internalization via endocytosis. Therefore, we propose that a key CD163 domain might interact and synergistically cooperate with MYH9 to facilitate PRRSV infection.

Using a co-IP assay we first confirmed that CD163 and MYH9 in PAMs interacted with one another regardless of PRRSV infection. Meanwhile, COS-7 cells, which express the non-muscle myosin II isoform MYH10 instead of MYH9 (Conti and Adelstein, 2008), were unable to support PRRSV infection even in the presence of porcine CD163. Notably, MYH9 shares a high level of amino acid sequence similarity with MYH10, with exception of the non-helical tail region located within the MYH9 C-terminal region (PRA) (Li et al., 2018). Therefore, the PRA region appears to be crucial for PRRSV infection and thus PRA was subsequently used to narrow down the CD163 interaction region to residues within the SRCR1-4 region of CD163. The direct interaction between SRCR1-4 and PRA was further confirmed by both far-Western blot and indirect ELISA analyses. Interestingly, as a cofactor involved in PRRSV infection, MYH9 homologs from various species (porcine or human origin) were shown to interact with porcine SRCR1-4, an observation also consistent with patterns of PRRSV permissiveness observed in cell lines of human origin toward PRRSV infection after introducing porcine CD163. Moreover, the different roles of SRCR1-4 or SRCR5-CT domains in the PRRSV replication cycle were found, whereby SRCR1-4 specifically played a critical role in PRRSV internalization to ensure overall viral replication efficiency.

Although MYH9 is mainly found within the cytoplasm, a fraction of MYH9 is also associated with the cellular membrane (Nebl et al., 2002; Xiong et al., 2015). Upon stimulation by PRRSV infection, CD163 and MYH9 have been observed to be up-regulated (Patton et al., 2009; Gao et al., 2016). Meanwhile, membrane re-distribution of MYH9 was observed if endocytosis was triggered by switching the temperature of virion-attached cells from 4°C to 37°C (Arii et al., 2010; Sun et al., 2014; Xiong et al., 2015). Therefore, these data imply that both initially membrane-associated MYH9 and membrane re-distributed MYH9 might participate in the PRRSV replication cycle. In this study, MYH9 within the cellular membrane directly interacted with recombinant SRCR1-4 in the absence of other adaptor proteins (as expected) and this interaction was first confirmed by the co-localization of SRCR1-4 with endogenous MYH9 in PAMs. We further demonstrated that incubation of recombinant SRCR1-4 with PAMs significantly diminished PRRSV internalization without affecting attachment, with subsequent and marked impairment of PRRSV replication. Moreover, SRCR1-4 protein conferred broad protection of PAMs against infection by both PRRSV-1 and PRRSV-2. Together, these data suggest that SRCR1-4 acted as a “competitive binding partner” that interrupted the direct CD163-MYH9 interaction during PRRSV internalization by permissive cells.

## Conclusion

In conclusion, our results provide the first direct evidence that CD163 and MYH9 act synergistically to mediate optimal internalization of PRRSV virions via the direct interaction between SRCR1-4 and MYH9 C-terminal domain (PRA). Recombinant SRCR1-4 achieved broad inhibition of virus replication (regardless of PRRSV genotype) via the interruption of the direct interaction between endogenous CD163 and MYH9 that is required for PRRSV internalization. Ultimately, this study provides new insights that enhance our understanding of PRRSV invasion mechanisms and suggests a potential antiviral strategy against PRRSV infection.

## Data Availability

All datasets generated for this study are included in the manuscript and/or the Supplementary Files.

## Author Contributions

GH and BX performed the research, analyzed the data, and drafted the manuscript. LL and YN contributed to the protein expression. LZ, KL, and QZ contributed to the construction of the cell lines. JH and JS contributed to the confocal immunofluorescence assay. CW, JW, and E-MZ conceived the study, carried out the additional analyses, and finalized the manuscript. All authors contributed to the revising of the manuscript.

## Conflict of Interest Statement

The authors declare that the research was conducted in the absence of any commercial or financial relationships that could be construed as a potential conflict of interest.

## Supplementary Material

FIGURE S1 Construction of the bicistronic plasmids expressing PRA and CD163 or its mutant. The internal ribosome entry site (IRES) sequence amplified from pTRIP-CMV-IRES-Puro wad cloned into the multiple cloning site of pCAGEN. The cDNAs of PRA and full length CD163 or mutants were inserted into bicistronic pCAGEN.

FIGURE S2 Characterization of recombinant CD163 SRCR1-4-His by 12% SDS–PAGE after purification. Lane 1, the CD163 SRCR1-4-His under reducing conditions. Lane 2, molecular-mass marker (labeled in kDa).

FIGURE S3 Recombinant SRCR1-4 protein not co-localized with MYH9 within the cytoplasm. PAMs were incubated with SRCR1-4 or the same quantity of BSA. Cells were permeabilized by Triton X-100 and subjected to immunofluorescence staining of MYH9 (red) and SRCR1-4 (green) and cellular nuclei were counterstained with DAPI (blue). The MYH9 and SRCR1-4 was visualized by confocal microscopy.

FIGURE S4 Protein expression level of the receptors or cellular factors. **(A)** Cells were cultured in 6-well plates, and the expression of MYH9, CD163, and Vimentin by cell lines was detected by Western blot. **(B)** Cells were cultured in 6-well plates, and the expression of MYH9, CD163, SRCR1-4, and SRCR5-CT by cell lines was detected by Western blot.
