# Supplementary figures and images for: Direct Interaction Between CD163 N-Terminal Domain and MYH9 C-Terminal Domain Contributes to Porcine Reproductive and Respiratory Syndrome Virus Internalization by Permissive Cells
Source: Front Microbiol. 2019 Aug 6;10:1815. doi: 10.3389/fmicb.2019.01815 (PMC6691103; doi:10.3389/fmicb.2019.01815)

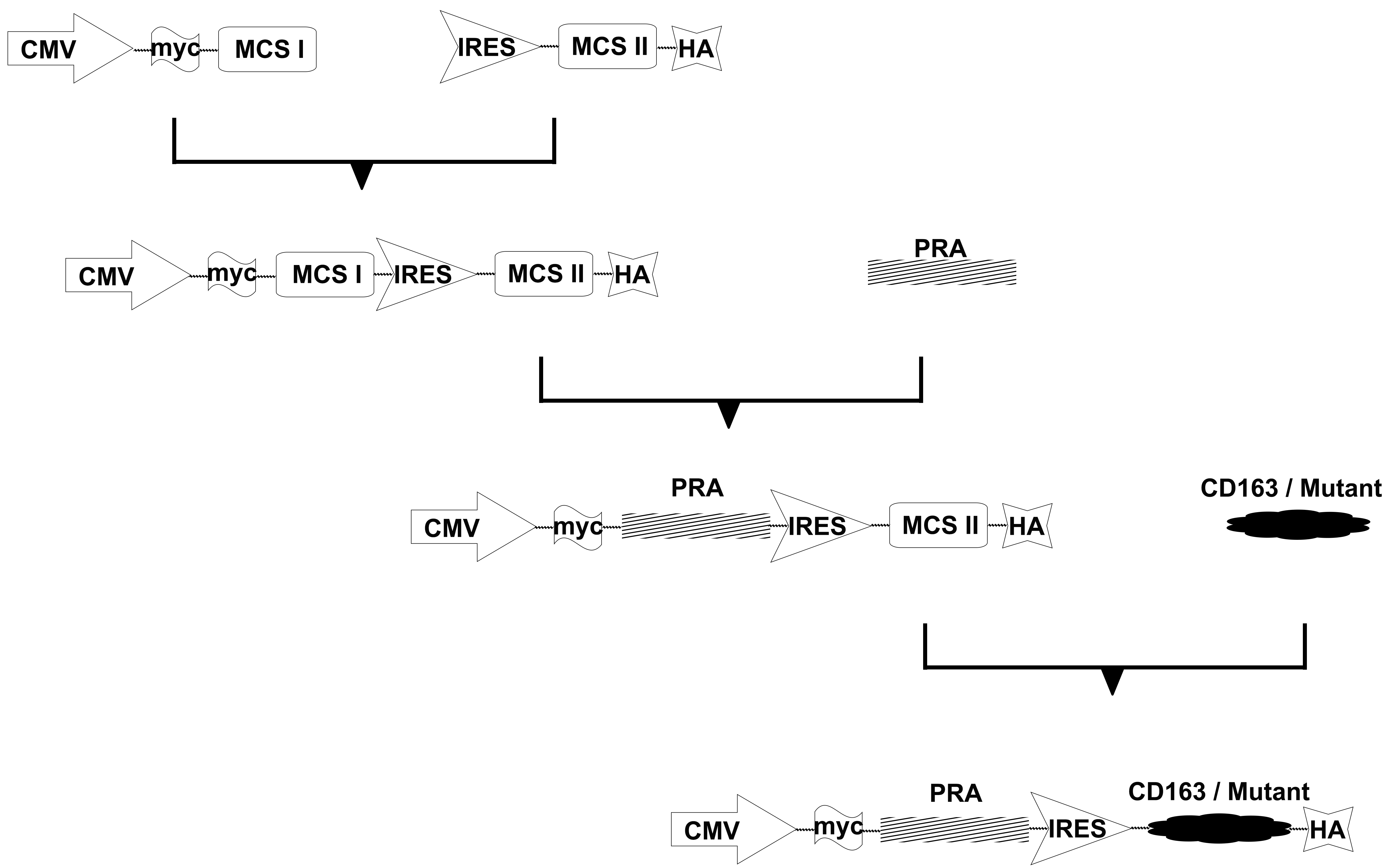

Supplement: FIGURE S1 — Construction of the bicistronic plasmids expressing PRA and CD163 or its mutant. The internal ribosome entry site (IRES) sequence amplified from pTRIP-CMV-IRES-Puro wad cloned into the multiple cloning site of pCAGEN. The cDNAs of PRA and full length CD163 or mutants were inserted into bicistronic pCAGEN. [file Image_1.TIF]

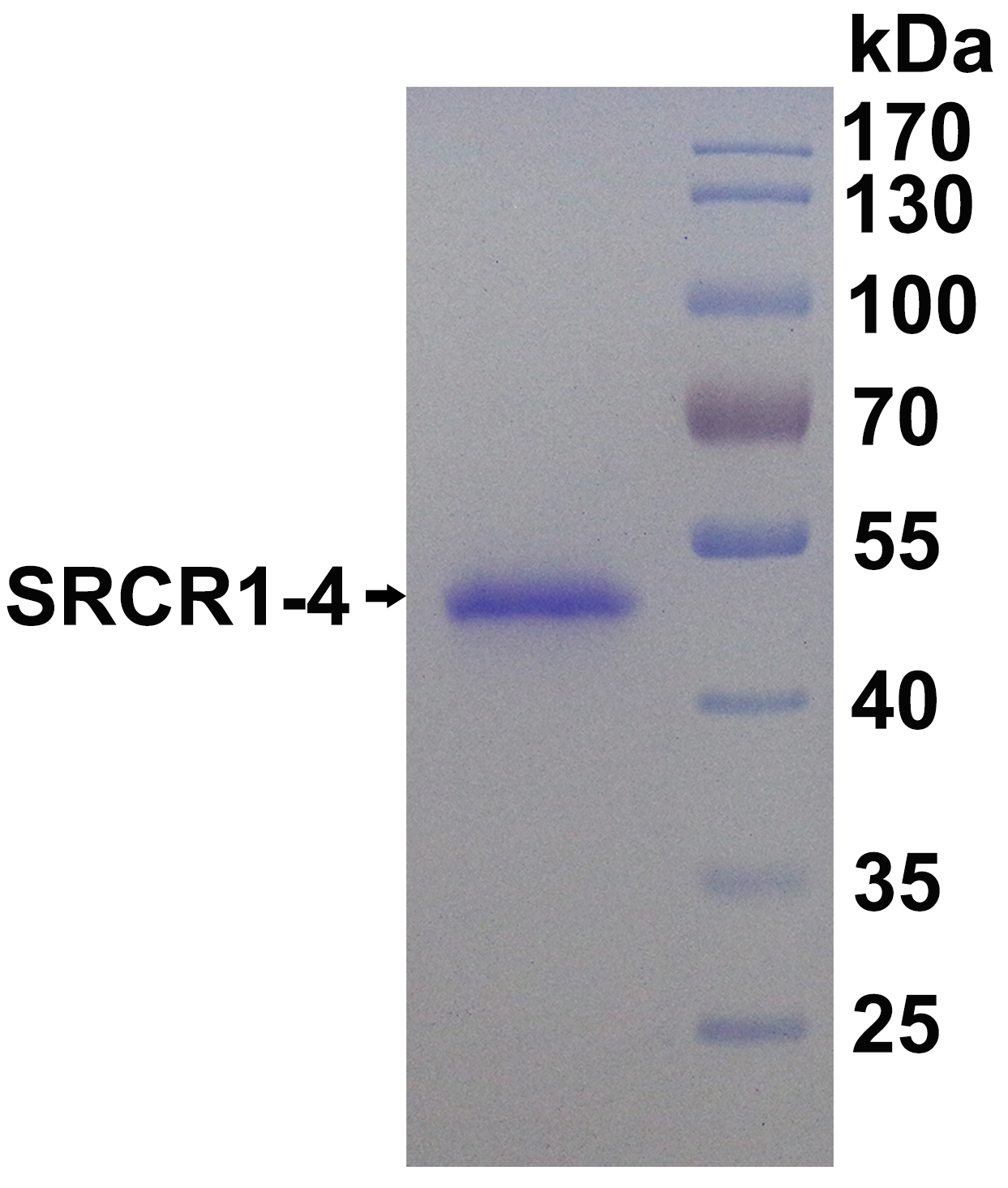

Supplement: FIGURE S2 — Characterization of recombinant CD163 SRCR1-4-His by 12% SDS–PAGE after purification. Lane 1, the CD163 SRCR1-4-His under reducing conditions. Lane 2, molecular-mass marker (labeled in kDa). [file Image_2.TIF]

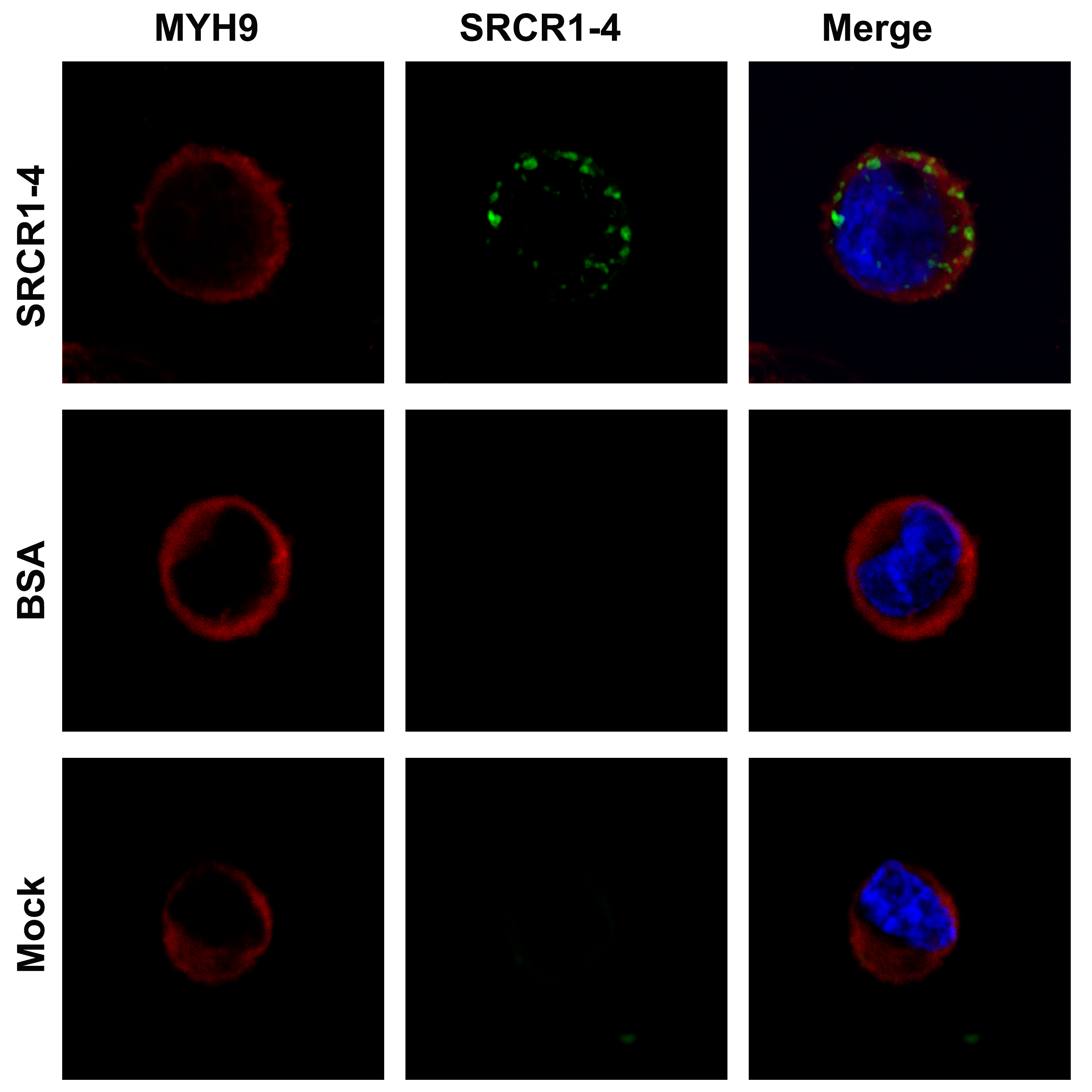

Supplement: FIGURE S3 — Recombinant SRCR1-4 protein not co-localized with MYH9 within the cytoplasm. PAMs were incubated with SRCR1-4 or the same quantity of BSA. Cells were permeabilized by Triton X-100 and subjected to immunofluorescence staining of MYH9 (red) and SRCR1-4 (green) and cellular nuclei were counterstained with DAPI (blue). The MYH9 and SRCR1-4 was visualized by confocal microscopy. [file Image_3.TIF]

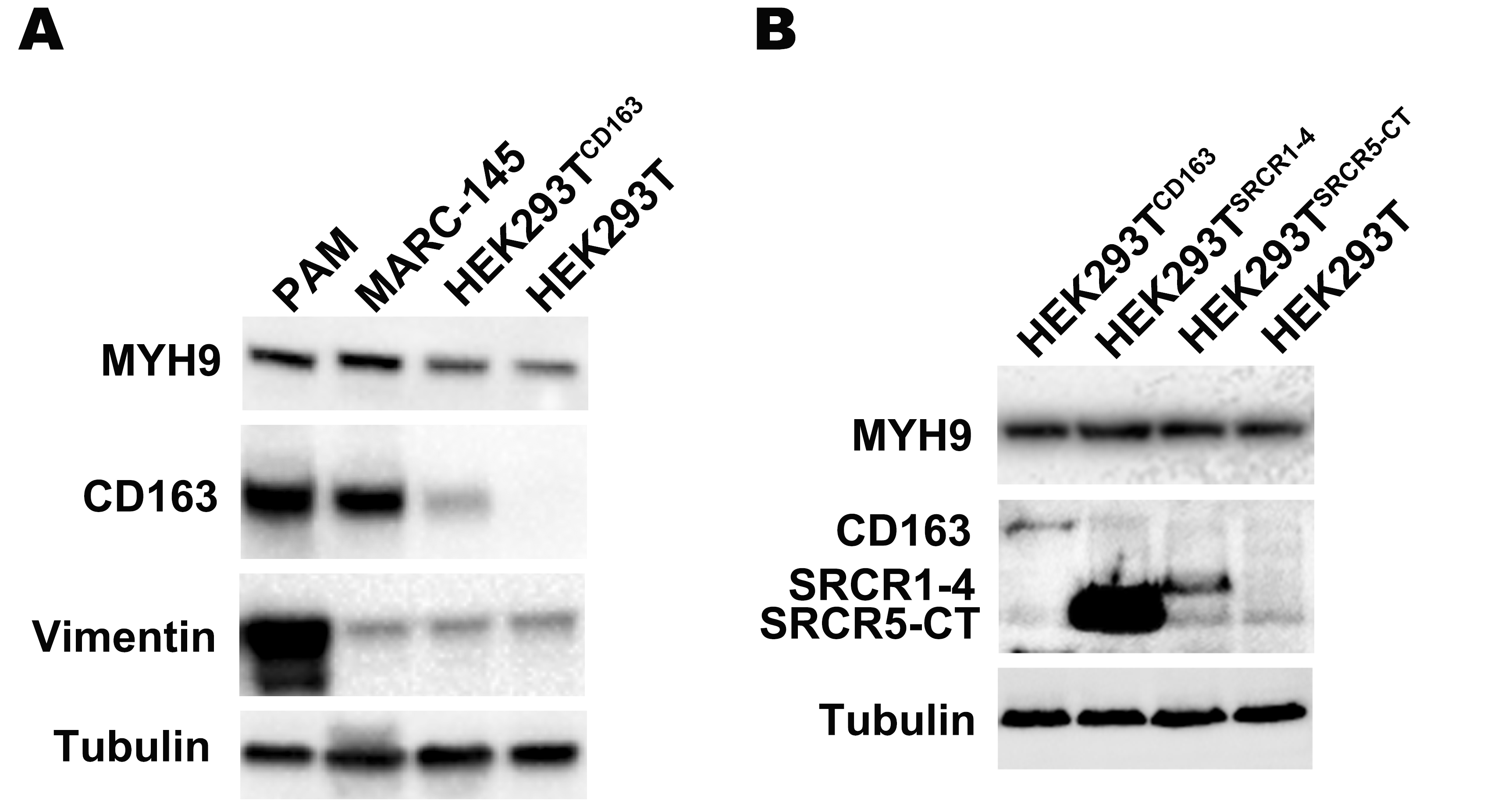

Supplement: FIGURE S4 — Protein expression level of the receptors or cellular factors. (A) Cells were cultured in 6-well plates, and the expression of MYH9, CD163, and Vimentin by cell lines was detected by Western blot. (B) Cells were cultured in 6-well plates, and the expression of MYH9, CD163, SRCR1-4, and SRCR5-CT by cell lines was detected by Western blot. [file Image_4.TIF]
